# Sea-level stands from the Western Mediterranean over the past 6.5 million years

**DOI:** 10.1038/s41598-020-80025-6

**Published:** 2021-01-21

**Authors:** Oana A. Dumitru, Jacqueline Austermann, Victor J. Polyak, Joan J. Fornós, Yemane Asmerom, Joaquín Ginés, Angel Ginés, Bogdan P. Onac

**Affiliations:** 1 Karst Research Group, School of Geosciences, University of South Florida, 4202 E. Fowler Ave., NES 107, Tampa, FL 33620 USA; 2 Biology and Paleo Environment Division, Columbia University, Lamont-Doherty Earth Observatory, Palisades, NY 10964 USA; 3 Department of Earth and Environmental Sciences, Columbia University, Lamont-Doherty Earth Observatory, Palisades, NY 10964 USA; 4 Department of Earth and Planetary Sciences, University of New Mexico, Albuquerque, NM 87131 USA; 5 Earth Sciences Research Group, Universitat de les Illes Balears, Ctra. Valldemossa km 7.5, 07122 Palma, Mallorca Spain; 6 Emil G. Racoviță Institute, Babeș-Bolyai University, Clinicilor 5-7, 400006 Cluj-Napoca, Romania

**Keywords:** Ocean sciences, Climate sciences, Palaeoceanography, Palaeoclimate

## Abstract

Sea-level reconstructions are important for understanding past ice sheet variability and its response to past and future warming. Here we present Neogene and Quaternary sea-level snapshots using phreatic overgrowths on speleothems (POS) from caves on Mallorca, Spain. POS are excellent sea level index points because of their clear relationship to sea level and precise U–Pb chronology. We find that local sea-level before and at the onset of the Messinian Salinity Crisis was at 33.3 ± 0.25 m (6.54 ± 0.37 Ma) and 31.8 ± 0.25 m (5.86 ± 0.60 Ma) above present levels, respectively. We further present global mean sea level (GMSL) estimates, i.e. local sea level corrected for glacial isostatic adjustment and long-term uplift, for three other POS. The results show that GMSL during the Pliocene–Pleistocene Transition was 6.4 m (− 2.0–8.8 m) at 2.63 ± 0.11 Ma and during the beginning and the end of the Mid-Pleistocene Transition was − 1.1 m (− 5.6–2.4 m) and 5 m (1.5–8.1 m), respectively. These estimates provide important constraints for the past evolution of sea level and show that local sea level prior to the MSC was similar to the highest stand during the Pliocene, with markedly lower position afterwards.

## Introduction

Accurate projections of future sea-level change rely on a thorough understanding of the mechanisms driving its complex spatio-temporal evolution^1^. The main causes of present global sea level rise are thermal expansion caused by warming of the oceans and increased melting of ice sheets and glaciers^2^. Hence, a large uncertainty for future sea-level change is the response of ice sheets to ongoing warming due to current and projected increases in atmospheric greenhouse gases^2,3^. Reconstructing past sea level changes during periods when Earth’s climate was warmer than today provides a window into ice sheet’s future behaviour and can contribute to more confident projections of rates of sea-level rise^3,4^. However, the limited number of robust sea level proxies, their sparse and irregular distribution, lack of absolute chronology, and frequent hiatuses in records, hinder our understanding of sea level changes over intervals older than the Last Interglacial, which is better documented^5,6^.

In this study we focus on sea level in the Mediterranean over the past 6.5 Myr (million years), which experienced changes due to Milankovitch cycles, continuous cooling into the present-day ice age, and was punctuated by the Messinian Salinity Crisis (MSC)^7,8^. The MSC is a major geological event during which a combination of glacio-eustatic, tectonic, and erosional processes, as well as climate variability, caused the Mediterranean Sea to repeatedly become partly to nearly desiccated^9–11^. While its timing has been relatively well established (5.97–5.33 Ma)^12,13^, the sea-level elevation before and at onset of the MSC remains highly uncertain^14–16^. The Messinian time period was followed by the Pliocene Epoch, which includes the Pliocene Climatic Optimum and the mid-Piacenzian Warm Period (MPWP), both intervals that have been considered as possible analogues for investigating ice sheet sensitivity in a warmer than present climate, since their atmospheric temperatures were ~ 4 and 2–3 °C, respectively, higher than preindustrial values^17,18^. The Pliocene–Pleistocene Transition (3.0–2.5 Ma)^19^ occurred when the Northern Hemisphere shifted from largely ice free conditions to extensive and repeated growth of the major ice sheets including the Laurentide and Fennoscandian ice sheets^20^. The passage from Lower to Middle Pleistocene, known as the mid-Pleistocene Transition (MPT, ~ 1.25–0.7 Ma) marks a shift from mostly symmetric 41-ka to strongly asymmetric 100-ka cycles^21,22^.

**Figure 1:**
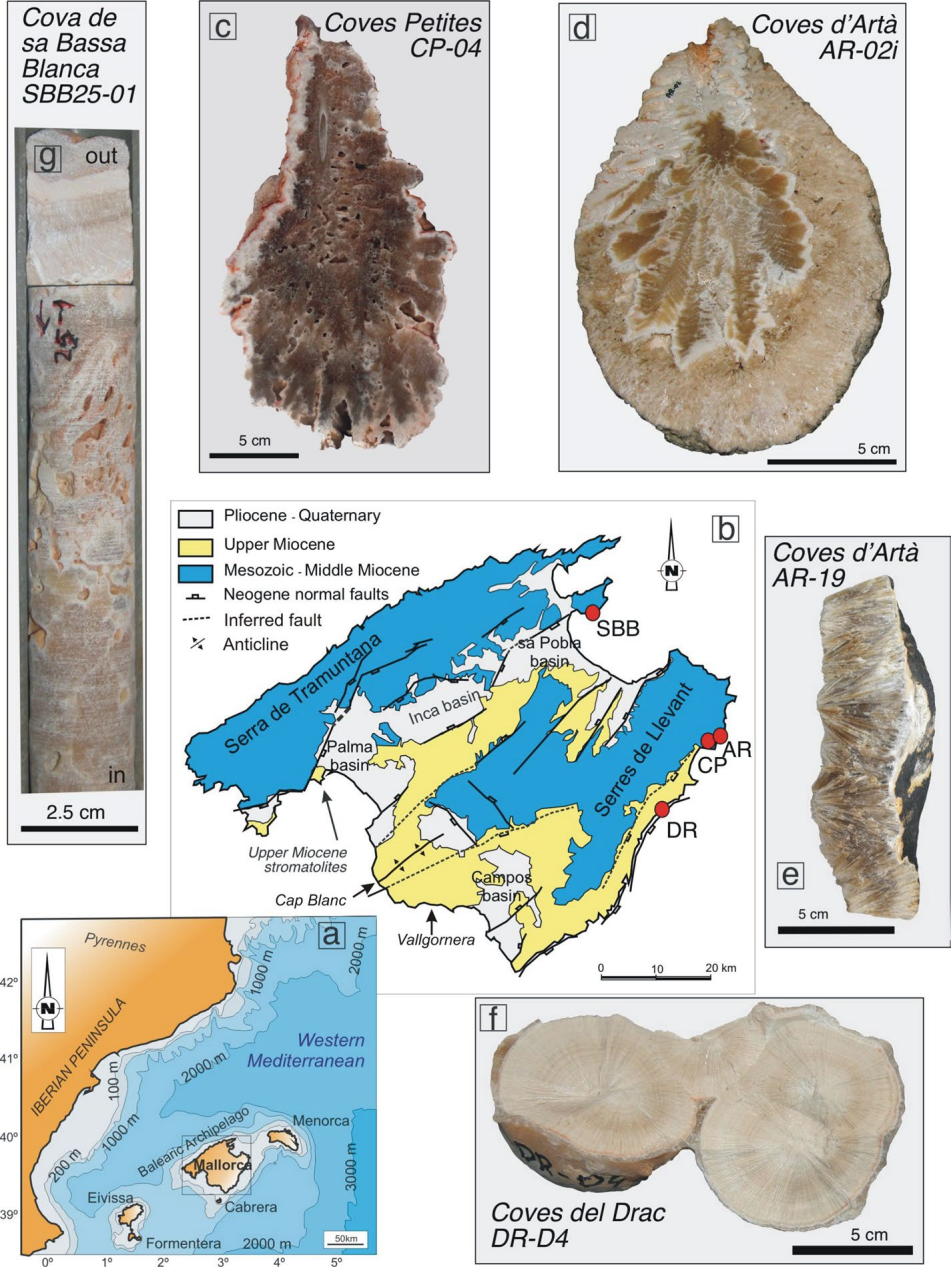
Sites location and samples. (**a**) Map of Mallorca in the western Mediterranean (gray rectangle). (**b**) Location of caves sampled for POS (red solid dots). (**c-g**) Cross-section of the investigated POS: (**c**) CP-04, (**d**) AR-02i, (**e**) AR-19, (**f**) DR-D4v, and (**g**) SBB25-01. Maps (**a**, **b**) are available under CC Public Domain License from https://pixabay.com/illustrations/map-europe-world-earth-continent-2672639/ and https://pixabay.com/illustrations/mallorca-map-land-country-europe-968363/, respectively, on which the bathymetry and geology were overlapped.

Sea level changes during these intervals have been reconstructed using a variety of geological proxies and techniques including benthic oxygen isotopes^23^, measurements of planktonic foraminifera and marginal basin residence time^24^, paleo-shorelines^25^ and back-stripped continental margins including interpretations of shallow-marine sediments^26,27^. Each approach to reconstructing Pliocene–Pleistocene global mean sea level has its advantages and disadvantages: records based on marine sediment cores are generally time continuous, however they lack absolute chronologies and a clear relationship to past sea level. Geologic records, such as paleo-shorelines, can be dated more directly but only constrain local sea level, not the global ice-equivalent mean. Local sea level can differ from global mean sea level (GMSL) due to uplift or subsidence of the solid Earth. Processes that cause such deformation include sediment loading/unloading^28^, dynamic topography^29,30^, tectonic deformation, and glacio-isostatic adjustment (GIA). GIA is the viscoelastic response of the crust, its gravity field, and rotation axis to changes in the ice and ocean load^31^. Corrections can be applied to account for these adjustments, but they can be highly uncertain. Hence, more quantitative spatial and temporal records are critical to better understand these processes and to provide better constraints of the GMSL. These GMSL reconstructions can help validating numerical ice sheet models that we are largely reliant on when projecting future sea-level changes^4,32^. Our work contributes to this line of research by providing snapshots of sea level still stands during those key time intervals mentioned above using U–Pb dated phreatic overgrowths on speleothems (POS) from littoral caves on the island of Mallorca, in the western Mediterranean (Fig. 1).

### POS as sea level index points

Documented in Mallorcan eastern and southern coastal caves, these phreatic deposits form in a particular geochemical environment in which pre-existing vadose speleothems (stalactites and stalagmites) become partly submerged in tidally-controlled brackish pools^33^. The POS form on submerged cave walls and vadose speleothems at and just below the water table and are comprised of well-preserved, densely crystallized calcite or aragonite (see “Methods”). Because the caves hosting POS are proximal to the coastline (within 300 m), the hydraulic gradient between them and the Mediterranean Sea is insignificant, thus, the brackish water table in these caves is today, and was in the past, coincident with sea level. As long as sea level remains at the same elevation for ~ 300 years or more, enough carbonate precipitation occurs within the tidal range at and few centimetres below the air–water interface to be sampled and dated^6^. Therefore, the presence of POS horizons at different elevations precisely marks the positions of paleo-water tables and consequently their associated sea level positions. The indicative range for POS is the vertical extent over which the carbonate encrustations occur, and the reference water level is zero at the widest part of the POS^34^. Ultimately, the POS morphology depends on the size, shape, and extent of immersion of the pre-existing vadose speleothems in the brackish lens^33^. Absolute chronology of POS can be obtained by using U–Pb method, since these deposits often contain suitable uranium and lead concentrations for accurate dating. Studies over the past decade have demonstrated the suitability of POS as meaningful sea-level index points^6,34,35^.

## Results and discussions

The results for each POS sample used in this study are listed in Table 1 (for detailed analytical descriptions and further information see “Methods” section).

**Table 1:** POS data and inferred GMSL estimates.

Cave name	Sample code	POS elevation (m)	Age (Myr)	GIA correction (m)	Uplift correction (m)	GMSL (m)
Coves Petites	CP-04	33.3	6.54 ± 0.37	–	–	–
Coves d’Artà	AR-02i	31.8	5.86 ± 0.60	–	–	–
	AR-19	14.3	2.63 ± 0.11	4.0 ± 2.1	5.4 (1.5 – 11.6)	6.4 (− 2.0–8.8)
Coves del Drac	DR-D4v	3.9	1.25 ± 0.09	2.4 ± 3.3	2.6 (0.7 – 5.5)	− 1.1 (− 5.6–2.4)
Cova de sa Bassa Blanca	SBB25-01	7.8	0.8 ± 0.16	1.0 ± 2.9	1.6 (0.5 – 3.5)	5.0 (1.5–8.1)

Measured elevations have an uncertainty of 0.25 m. Age uncertainties are reported as 2σ absolute values. The GIA correction uncertainty is 1σ. Uplift correction shows the median value and the 16th and 84th percentiles in parentheses as uncertainty bounds. The GMSL estimates include correction for GIA and long-term uplift and show the mode and the 16th and 84th percentiles in parentheses as uncertainty bounds.

### Sea-level snapshots before and at the onset of MSC

**Figure 2:**
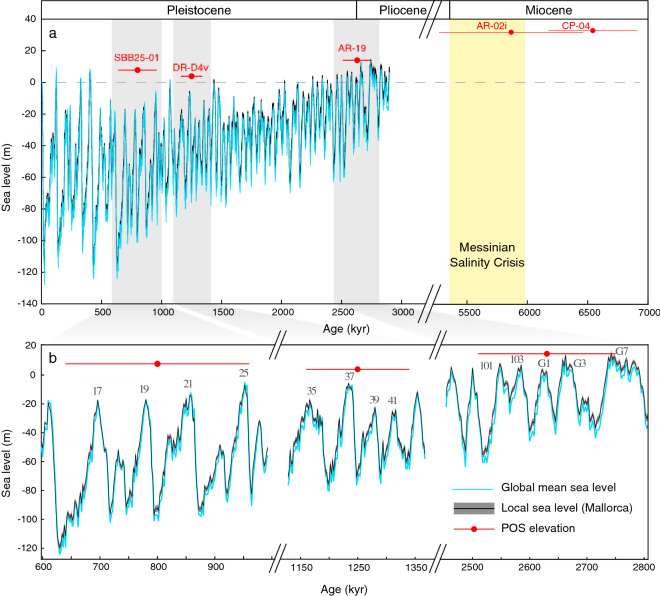
Local sea level at Mallorca. (**a**) Plot shows the whole temporal range. (**b**) Enlarged views for the three most recent data points. GMSL used in the GIA calculation is shown by the cyan line and based on the benthic oxygen isotope stack^20^. The resulting local sea level is shown by the black line. Uncertainty in local sea level that arises from uncertainties in the Earth structure is shown as a gray band around the black line (1σ). Elevation of inferred local sea level for five POS presented here is shown in red, age uncertainties are 2σ. Gray numbers and letters in panel **b** denote Marine Isotope stages.

Sample CP-04, collected from Coves Petites (Eastern Mallorca; Fig. 1b,c), marks a sea level stand currently at 33.3 ± 0.25 m higher than present corresponding to the Mid-Messinian at 6.54 ± 0.37 Ma, before the onset of the Messinian Salinity Crisis (Fig. 2). Evidence of even higher sea level before the MSC is also provided by the Reef Complex Unit (Upper Tortonian) exquisitely exposed in vertical sea-cliff outcrops between Vallgornera and Cap Blanc in southern Mallorca^36^ (see Fig. 1b for locations). The top of this unit lies at about 70 m above present sea level^36^, but the outcropping stratigraphic geometries and facies distribution indicate several cyclic fluctuations in the position of the reefal facies, all interpreted to have resulted from sea-level oscillations^37^. The most recent K–Ar dates on biotites from the back-lagoon deposits near Cap Blanc indicate ages of 6.45 and 6.23 Ma^38^, but since there are several complete sequences of reefal progradation located at different levels, a better chronostratigraphic constraint of this unit is required to compare it to our cave deposit. Additionally, despite the flat appearance of this platform, a wide and gentle anticline with a NE-SW axis direction related to the movement of Palma and Campos NE-SW normal faults is suggested^39^ (Fig. 1b). This means that at present, the reef location could be higher than our Coves Petites POS due to this tectonic deformation. Pre-MSC indirect evidence of sea level from the same island is also provided by outcropping microbial build-up (stromatolites) in the Porto Pi area at the western end of the Palma Harbour (Fig. 1b). These deposits, reported as Upper Miocene, belong to the Santanyí Limestone and developed in a shallow marine environment (between 0 and 10 m) during periods of limited connection with open-marine realm, prompting evaporative and hyper-saline conditions^40^.

Sample AR-02i, currently at 31.8 ± 0.25 m higher than today (Fig. 2a), comes from Coves d’Artà (400 m east of Coves Petites), which hosts seven Pliocene sea-level stands^34^. A cross section of AR-02 collected from the highest horizon of this cave indicates two distinct phreatic overgrowth deposition events (see Fig. 1d). The outer layer (AR-02) precipitated at 4.39 ± 0.39 Ma^34^, whereas the inner part (AR-02i) yielded an older age of 5.86 ± 0.60 Ma (Table 1). AR-02i overlaps with an early phase of the MSC (5.97–5.61 Ma) known as the Primary Lower Gypsum stage^13^. The age errors of AR-02i unfortunately do not clearly support or exclude any of the proposed scenarios for the onset or termination of the MSC^16^.

The horizons marked by samples CP-04 and AR-02i provide directly dated sea level markers back to the Messinian Stage. We highlight that the elevations reported here are local sea level, which differs from GMSL due to glacial isostatic adjustment and long-term deformation. Most notably, evaporite deposition during the MSC can cause loading of the crust and noticeably affect local sea level. Correcting for this effect requires quantifying the spatio-temporal loading associated with these sediments and modelling the respective sea level response, which is beyond the scope of this paper (see further justification in the “Methods” section). Hence, we do not attempt to relate local sea level to its global mean for these two samples. Still, these two POS show clear evidence of sea level stands higher than today that persisted long enough to allow the precipitation of the carbonate encrustations.

### Pliocene–Pleistocene sea-level snapshots

For the samples postdating the MSC we translated the local sea-level observations into GMSL by correcting for GIA and long-term deformation, as explained in the “Methods” section. Figure 2 shows a GMSL prediction based on benthic oxygen isotope stack^20^ and the resulting local sea level on Mallorca (accounting for GIA) along with the elevation of local sea level indicators.

Six of the POS-derived sea-level still stands in Artà were already reported^34^ and include the notable samples AR-02 and AR-03. The former, which has an age of 4.39 ± 0.39 Ma, yielded a GMSL estimate of 25.1 m (10.6–28.3 m) above present sea level and formed within the warmest interval of the Pliocene (~ 4.4 to 4.0 Ma). Sample AR-03 with an inferred GMSL of 17.4 m (6.8–20.3 m) higher than today documents a horizon with an age of 3.27 ± 0.12 Ma that likely formed at the onset of the MPWP.

**Figure 3:**
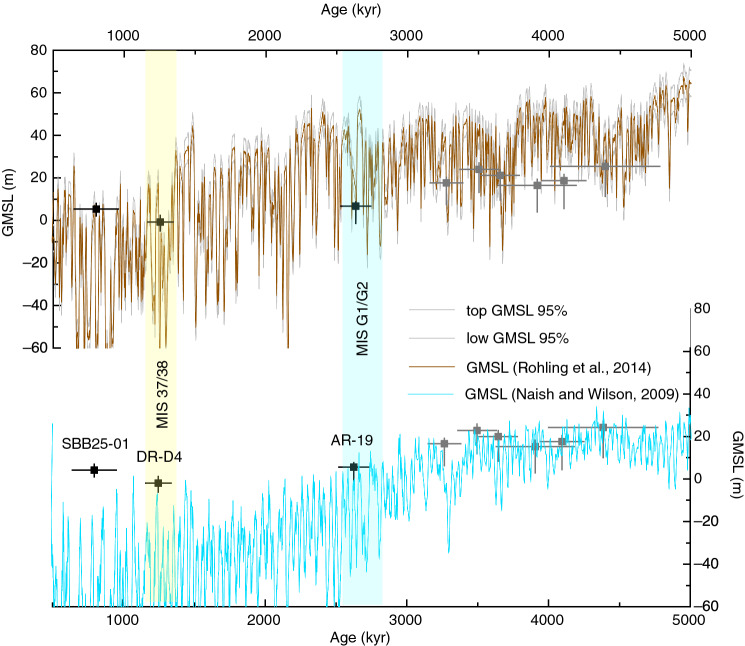
Pliocene and Pleistocene global mean sea level estimates. POS-derived Pliocen and Pleistocene GMSL are indicated by silver^34^ and black (this study) markers, respectively (age uncertainties are 2σ; the GMSL of the marker corresponds to the mode and the error bars to the 16th and 84th percentiles). Thermal expansion correction is not applied for any of these estimates. Brown and cyan curves show two oxygen isotope based sea level reconstructions (brown is the GMSL derived estimates from the planktonic foraminifera and the marginal basin residence time^24^ and cyan is the GMSL curve derived from the conversion of LR04 benthic foraminiferal δ^18^O stack by scaling with a calibration of 0.011 ppt m^−1^ as in^23^).

A new POS level currently located at 14.3 m higher than present sea level has recently been identified in Artà; sample AR-19 (Fig. 1e) from this horizon yielded an age of 2.63 ± 0.11 Ma, overlapping with the Pliocene–Pleistocene transition. This is concomitant with an increase in benthic δ^18^O values at 2.64 Ma (Fig. 3; the MIS G1/G2 boundary^20^). POS AR-19 yielded a GMSL of 6.4 m (− 2.0–8.8 m) after its elevation was corrected for GIA and long-term uplift (Table 1). A tentative interpretation of this sea level stand, which is lower than all other Pliocene POS existing in Artà (Table 1), is that it formed during an early Pleistocene interstadial (Fig. 2b). This interpretation is in line with GMSL estimates that are based on the benthic stack (e.g.^23^, Fig. 3). The warm MPWP was followed by a cooling into the Pleistocene with an intensification of Northern Hemisphere glaciations around ~ 2.7 Ma^41^. If this POS did indeed form during an interstadial, it implies that interglacial sea level markedly decreased after the MPWP. Comparing our results to the GMSL reconstruction by Rohling et al.^24^, however, tells a different story: the AR-19 estimate aligns with sea level during a stadial in their reconstruction and might therefore record a sea level low stand rather than a high stand. While we consider this interpretation less likely, we emphasize that our data alone only record sea level still stands, and we are therefore not able to discriminate whether they occurred during a stadial or an interstadial. We note that for the Pliocene, however, we did speculate that the POS formed during interstadials, rather than stadials^34^.

Two other samples, DR-D4 and SBB25-01, precipitated during the transition from Lower to Middle Pleistocene. DR-D4 (Fig. 1f) was recovered from a well-defined sea-level horizon located at 3.9 m above present sea level in Coves del Drac (Fig. 1b). The sample gave a U–Pb age of 1.25 ± 0.09 Ma, which is coincident with the onset of MPT, marked by the shift between MIS 38 and MIS 37^42^ (Fig. 2). An overall cooling trend during the MPT is exhibited in the deep ocean temperature, derived from benthic foraminiferal Mg/Ca ratios from the North Atlantic^43^. Similar trends are suggested by the sea ice reconstruction based on ice biomarker IP25 from Site U1343 in the Bering Sea^44^, and by the record of dust and iron supply into the Southern Ocean^45^, concomitant with a substantial increase in sea ice extent^21^. The GMSL estimate obtained from DR-D4 is − 1.1 m (− 5.6–2.4 m), which falls within the stadial-interstadial range of Rohling et al.^24^ during this time, and is slightly higher than the interstadial estimate by^23^ for MIS 37, as shown in Fig. 3.

Sample SBB25-01 (Fig. 1g) from Cova de Sa Bassa Blanca in the northern part of the island (Fig. 1b) formed at an elevation of 7.8 m. This is the first cave in which POS were observed and tentatively interpreted to represent past Pleistocene sea stands^46^. Although the cave hosts over 15 POS horizons, sampling activities are problematic due to high CO_2_ levels (> 2–4%) in the cave atmosphere. The GMSL of 5 m (1.5–8.1 m) higher than present at 0.8 ± 0.16 Ma obtained from sample SBB25-01 is in agreement with the reconstruction by Rohling et al.^24^, and indicates that it formed during an interstadial, possibly MIS 19. Assuming this, its GMSL range is 25 m higher than the estimates of^23^ for this period (Fig. 3). While this could indicate a difficulty in interpreting GMSL from the benthic stack, we note that Sa Bassa Blanca Cave is furthest away from Coves d’Artà (Fig. 1b), from which data were used to constrain the uplift rate. It is therefore possible that spatial trends in long-term uplift could have affected our GMSL estimate at this site.

## Conclusions

Our results document the position and timing of sea level since the Upper Miocene until the Middle Pleistocene, overlapping key time intervals for which global mean sea-level estimates are still highly uncertain. The sea level index points represented by samples CP-04 and AR-02i in the Upper Miocene show that local sea level before and near the onset of the MSC was 33.3 ± 0.25 m and 31.8 ± 0.25 m above present sea level, respectively. While these elevations are not corrected for GMSL (no GIA and long-term uplift corrections were applied), they may offer starting points for assessing whether sea-level drawdown in the western Mediterranean happened gradually or rapidly. Sea-level elevations documented by samples of Pliocene and Pleistocene ages were translated into GMSL. As a result, the new GMSL of 6.4 m (− 2.0–8.8 m) estimated for the Pliocene–Pleistocene Transition at 2.63 ± 0.11 Ma, indicates an important lowering of GMSL immediately after the Pliocene. Also, new GMSL estimates of − 1.1 m (− 5.6–2.4 m) and 5 m (1.5–8.1 m) at 1.25 ± 0.09 Ma and 0.8 ± 0.16 Ma, respectively, correspond to the beginning and the end of Lower to Middle Pleistocene Transition period.

Our estimates are important snapshots of sea level still stands, but additional sea level index points will be useful to yield more context for our results. By providing direct estimates of sea level using POS as robust proxies, this work advances our understanding of sea level position during several past warm periods. These results therefore contribute to efforts of studying past warm periods to gain insight into the magnitude and frequency of sea level rise.

## Methods

### POS preservation, sampling, and U–Pb chronology

Based on current observations we know that the upper 40 cm of the water column is supersaturated with respect to calcium carbonate allowing for POS to form^47^. In some Mallorcan caves corrosion of carbonate minerals (calcite or aragonite) was noticed particularly when approaching the halocline, which occur at different depths depending on the distance of the cave from coast, seasonal meteoric recharge, and local lithological settings. However, at least one other study found both calcite and aragonite precipitating in the mixing zone, where numerical model predicted dissolution^48^. It appears that the inorganically precipitated carbonate POS are less sensitive to recrystallization and neomorphism (wet polymorphic transformation of aragonite to calcite) in these cave microenvironments. Since many of the investigated POS are entirely of aragonite mineralogy, this is a reliable indicator of the good preservation of our samples, considering they are millions of years old. Any significant diagenetic process would have altered the aragonite to calcite. Furthermore, the POS occurring at the highest elevations have never been submerged, or at least there is no evidence of higher sea level stands after their deposition. The same holds true for the lowermost POS in Sa Bassa Blanca and Drac caves. At no other point in time in the last 1.25 Ma was sea level in Mallorca 10 m or more above present sea level to cause an immersion of the POS to depths that would have placed them in potentially corrosive brackish water. Another evidence that the samples have not undergone diagenesis come from the uranium isotopic data of the samples. The δ^234^U values of the samples is a good indication that the samples have not suffered diagenesis. The δ^234^U values of the old samples have secular equilibrium (~ 0), within error (Table 1). This evidence is remarkable for such old aragonite samples. Given uranium mobility in oxidizing waters, slight alteration results in change in δ^234^U.

POS samples were collected from horizons at different elevations in four caves (see Fig. 1b). The elevations were measured using a SUUNTO optical clinometer and a BOSCH DLE 50 Professional laser distance meter, which leads to an elevation uncertainty of 0.25 m. Due to the large thickness of some of the horizons, cores were drilled using a commercial cordless hand-held Hilti rotary hammer drill; otherwise, the whole POS was collected, as shown in Fig. 1.

Sub-samples of well-crystalized aragonite or calcite were cleaned and subjected to the conventional isotope dilution anion exchange resin chemistry for elemental separation. Next, they were dissolved in 15 N nitric acid and spiked with a mixed solution of ^229^Th–^233^U–^236^U–^205^Pb for obtaining Th, U and Pb isotopic ratios for uranium-series (U-series) and U/Pb chronology. U and Pb ratios were measured using a Thermo Neptune multi-collector inductively coupled mass spectrometer coupled with the Cetac Aridus II desolvating nebulizer at the Radiogenic Laboratory of the University of New Mexico in Albuquerque. Details on the analytical methodology have been previously reported^34,49^. Reduction of data is performed using PBDAT^50^ and ISOPLOT^51^. To correct the ages for the initial disequilibrium, an initial ^234^U/^238^U (δ^234^U_i_) activity of 1.75 was used, based on inferences of samples ranging from Holocene to Pleistocene dated using the U–Th method^6,34^. The ages were calculated using the U–Pb Concordia-constrained linear three-dimensional isochron, which contains the most complete information on the concordance between the two decay schemes and common Pb (see Supplementary Table S1).

### GIA modelling

To calculate a GIA correction for the samples postdating the MSC we use the gravitationally self-consistent sea level theory described in^52^ and^53^. This theory calculates the Maxwell viscoelastic response of Earth’s interior and its gravity field to spatiotemporal changes in ice and ocean load. It accounts for the migration of shorelines and the feedback into Earth’s rotation axis. On input, the GIA model requires the evolution of past ice sheets and Earth’s internal viscoelastic structure. We use the ice model ICE-6G by^54^ for the ice sheet evolution since 26 ka. Prior to this date, we base our GMSL reconstruction on the benthic oxygen isotope stack by^20^ and scale the stack to result in a GMSL low stand of − 120 m at 18 ka, which results in a scaling of 0.0112. We assume that 75% of the signal is driven by ice volume and the remaining 25% by temperature variations. For the time interval before 26 ka, we prescribe an ice geometry that matches the last deglacial geometry with the same GMSL value. For time intervals with a GMSL value above zero we uniformly melt the Greenland and West Antarctic ice sheets before uniformly melting the East Antarctic ice sheet. Changes in GMSL are assumed to be directly ice equivalent with a fixed oceanic area of 71.1%, a water density of 1000 kg m^−3^, and an ice density of 920 kg m^−3^. For Earth’s internal viscoelastic structure we assume a set of 36 different structure profiles that vary in lithospheric thickness (48 km, 71 km, and 96 km), upper-mantle viscosity (3 × 10^20^ Pa s and 5 × 10^20^ Pa s), and lower-mantle viscosity (3 × 10^21^, 5 × 10^21^, 7 × 10^21^, 10 × 10^21^, 20 × 10^21^, and 30 × 10^21^ Pa s). For the elastic and density structure we assume values from the seismic reference model PREM^55^.

The GIA model returns local sea level on Mallorca over the time period modelled here. The GMSL curve and the range of local sea level on Mallorca along with the elevation of local sea level indicators are shown in Fig. 2. To calculate the GIA correction for each POS we first take the GMSL reconstruction that spans the respective 2 σ age range and only consider high GMSL values during this time period. This selection is based on the assumption that the most favourable conditions for POS to form are during intermediate and warm periods. We acknowledge that if the POS formed during low stands, it would weaken this assumption and it would lead to a slightly larger uncertainty in the GIA correction. Specifically, we note all GMSL values that fall above the 90th percentile (of all GMSL values within this time range). For example, for sample SBB25-01 these are GMSL values above − 24 m. We then calculate the GIA correction for these values, which is the local sea level minus the GMSL. We do this for all 36 ice age model simulations to estimate a range of possible GIA corrections for each POS, of which we report the mean and one standard deviation. If we do not restrict that the POS formed during warm periods but instead consider that it could have formed during any time, the GIA correction becomes more positive with an increased uncertainty.

We do not attempt to relate local sea level to its global mean for the POS that record sea level prior to or near the MSC as justified in the next section. Therefore, GIA correction for these indicators were not calculated.

### Long-term uplift correction

The overall structure of Mallorca comprises a set of NE-SW trending basins and ranges, which are the result of complex tectonic movements that involve (i) a pre-orogenic Mesozoic extensional stage, (ii) a contractional Oligocene–Miocene orogenic stage, and (iii) a relative quiescent, mainly extensional, Late Miocene to recent post-orogenic stage^56^. The different basins and ranges are built up by thrust faults and associated folds, as shown in Fig. 1b ^56,57^ and the boundary between these major units are Upper Miocene normal faults^58^. In addition to this potential tectonic deformation, uplift or subsidence over the Pliocene–Pleistocene can be caused by mantle convection or sediment loading/unloading (e.g.,^59–61^).

When attempting to reconstruct ice equivalent GMSL we need to correct for these effects. The amount of local uplift at Coves d’Artà has been estimated by Dumitru et al.^34^ based on the observed elevation of six Pliocene sea level indicators and it was found that the median uplift rate at this site is 2.0 m/Myr (0.6–4.4 m/Myr; uncertainties constitute the 16th and 84th percentiles of the probability density function for the uplift rate). We use this uplift rate to correct the three most recent sea level indicators, assuming that the uplift is constant in time and across the island, where especially the later assumption might be challenged.

The POS that formed before or during the MSC could have been significantly affected by sediment deposition and the subsequent loading processes. Although the hydrology and salinity evolution of the Mediterranean basin during the MSC is well known, the budget of the evaporitic sedimentation is less well understood, since no complete series of the abyssal evaporites have been mapped yet^62^. Estimates of the total mass of salt deposited during the MSC exist (e.g. 1.44 × 10^18^ kg in the Western Basin and 6.44 × 10^18^ kg in the Eastern Basin^62^), but vary across the literature^63^. Additionally, the timing, but more importantly, the geographic distribution over which the deposits extend is uncertain, and so is the hydro-isostatic effect of the quasi-desiccation and rapid reflooding of the Mediterranean Sea^64^. Fully assessing the potential loading effect of these sediments as well as the GIA signal associated with time-dependent water level changes^9^ may be possible but beyond the scope of this work. We therefore resort to only reporting our estimates of uncorrected relative sea level for the older samples, CP-04 and AR-02i.

## Supplementary Information

Supplementary Legend.

Supplementary Table S1.

## Data Availability

All data generated or analyzed during this study are included in this published article.

## Code availability

The computer code used to do the sea-level (GIA) calculation, written in MATLAB, is available on github: https://github.com/jaustermann/SLcode.

## References

[1] Horton BP (2018). Mapping sea-level change in time, space, and probability. Annu. Rev. Environ. Resour..

[2] IPCC (2013). Climate Change 2013: The Physical Science Basis. Contribution of Working Group I to the Fifth Assessment Report of the Intergovernmental Panel on Climate Change.

[3] Fischer H (2018). Palaeoclimate constraints on the impact of 2 °C anthropogenic warming and beyond. Nat. Geosci..

[4] DeConto RM, Pollard D (2016). Contribution of Antarctica to past and future sea-level rise. Nature.

[5] Kopp RE, Simons FJ, Mitrovica JX, Maloof AC, Oppenheimer M (2009). Probabilistic assessment of sea level during the last interglacial stage. Nature.

[6] Polyak VJ (2018). A highly resolved record of relative sea level in the western Mediterranean Sea during the last interglacial period. Nat. Geosci..

[7] Hsü KJ, Cita MB, Ryan WBF (1973). The origin of the Mediterranean evaporates. Init. Rep. Deep Sea Drilling Proj..

[8] Roveri M (2014). The Messinian salinity crisis: Past and future of a great challenge for marine sciences. Mar. Geol..

[9] Coulson S (2019). The role of isostatic adjustment and gravitational effects on the dynamics of the Messinian salinity crisis. Earth Planet. Sci. Lett..

[10] Garcia-Castellanos D, Villaseñor A (2011). Messinian salinity crisis regulated by competing tectonics and erosion at the Gibraltar arc. Nature.

[11] Gargani J, Rigollet C (2007). Mediterranean sea level variations during the Messinian salinity crisis. Geophys. Res. Lett..

[12] Krijgsman W, Hilgen FJ, Raffi I, Sierro FJ, Wilson DS (1999). Chronology, causes and progression of the Messinian salinity crisis. Nature.

[13] Manzi V (2013). Age refinement of the Messinian salinity crisis onset in the Mediterranean. Terra Nova.

[14] Mas G (2018). Terrestrial colonization of the Balearic Islands: New evidence for the Mediterranean sea-level drawdown during the Messinian Salinity Crisis. Geology.

[15] Ohneiser C (2015). Antarctic glacio-eustatic contributions to late Miocene Mediterranean desiccation and reflooding. Nat. Commun..

[16] Pérez-Asensio JN, Aguirre J, Jiménez-Moreno G, Schmiedl G, Civis J (2013). Glacioeustatic control on the origin and cessation of the Messinian salinity crisis. Glob. Planet. Change.

[17] Dutton A (2015). Sea-level rise due to polar ice-sheet mass loss during past warm periods. Science.

[18] Fedorov AV (2013). Patterns and mechanisms of early Pliocene warmth. Nature.

[19] Tan N (2018). Dynamic Greenland ice sheet driven by pCO_2_ variations across the Pliocene Pleistocene transition. Nat. Commun..

[20] Lisiecki LE, Raymo ME (2005). A Pliocene-Pleistocene stack of 57 globally distributed benthic δ^18^O records. Paleoceanography.

[21] Clark PU (2006). The middle Pleistocene transition: Characteristics, mechanisms, and implications for long-term changes in atmospheric pCO_2_. Quatern. Sci. Rev..

[22] Pisias NG, Moore TC (1981). The evolution of Pleistocene climate: A time series approach. Earth Planet. Sci. Lett..

[23] Naish TR, Wilson GS (2009). Constraints on the amplitude of Mid-Pliocene (3.6–2.4 Ma) eustatic sea-level fluctuations from the New Zealand shallow-marine sediment record. Philos. Trans. R. Soc. A.

[24] Rohling EJ (2014). Sea-level and deep-sea-temperature variability over the past 5.3 million years. Nature.

[25] Miller KG (2012). High tide of the warm Pliocene: Implications of global sea level for Antarctic deglaciation. Geology.

[26] Miller KG (2020). Cenozoic sea-level and cryospheric evolution from deep-sea geochemical and continental margin records. Sci. Adv..

[27] Grant GR (2019). The amplitude and origin of sea-level variability during the Pliocene epoch. Nature.

[28] Moucha R, Ruetenik GA (2017). Interplay between dynamic topography and flexure along the US Atlantic passive margin: Insights from landscape evolution modeling. Glob. Planet. Change.

[29] Rowley DB (2013). Dynamic topography change of the Eastern United States since 3 million years ago. Science.

[30] Austermann J, Mitrovica JX, Huybers P, Rovere A (2017). Detection of a dynamic topography signal in last interglacial sea-level records. Sci. Adv..

[31] Mitrovica JX, Milne GA (2003). On post-glacial sea level: I. General theory. Geophys. J. Int..

[32] de Boer B, Haywood AM, Dolan AM, Hunter SJ, Prescott CL (2017). The transient response of ice volume to orbital forcing during the warm Late Pliocene. Geophys. Res. Lett..

[33] Ginés, J., Ginés, A., Fornós, J.J., Tuccimei, P., Onac, B.P., Gràcia, F. in *Mallorca: A Mediterranean benchmark for Quaternary studies* Vol. 18 (eds. Ginés et al.) 111–146 (Monografies de la Societat d’Història Natural de les Balears, 2012).

[34] Dumitru OA (2019). Constraints on global mean sea level during Pliocene warmth. Nature.

[35] Tuccimei P (2012). Decoding last interglacial sea-level variations in the western Mediterranean using speleothem encrustations from coastal caves in Mallorca and Sardinia: A field data: Model comparison. Quatern. Int..

[36] Pomar, L. & Ward, W. C. in *Sequence stratigraphy and depositional response to eustatic, tectonic and climatic forcing* (ed B.U. Haq) 87–112 (Springer, New York, 1995).

[37] Pomar, L., Ward, W.C., Green, D.G. in *Models for carbonate stratigraphy from Miocene reef complexes of the Mediterranean regions* Vol. 5 (eds Franseen et al.) 191–225 (Society of Economic Paleontologists and Mineralogists, Concepts in Sedimentology and Paleontology Series, 1996).

[38] Pomar L, Bassant P, Brandano M, Ruchonnet C, Janson X (2012). Impact of carbonate producing biota on platform architecture: Insights from Miocene examples of the Mediterranean region. Earth Sci. Rev..

[39] Gómez-Pujol L, Gelabert B, Fornós JJ, Rosselló VM, Pardo JE, Segura F, Onac BP (2013). Structural control on the presence and character of Calas: examples from Balearic Islands limestone rock macroforms. Geomorphology.

[40] Suarez-Gonzalez P, Arenas C, Benito MI, Pomar L (2019). Interplay between biotic and environmental conditions in pre-salt Messinian microbialites of the western Mediterranean (Upper Miocene, Mallorca, Spain). Palaeogeogr. Palaeoclimatol. Palaeoecol..

[41] Herbert TD, Ng G, Cleaveland Peterson L (2015). Evolution of Mediterranean sea surface temperatures 3.5–1.5 Ma: Regional and hemispheric influences. Earth Planet. Sci. Lett..

[42] Tzedakis PC, Crucifix M, Mitsui T, Wolff EW (2017). A simple rule to determine which insolation cycles lead to interglacials. Nature.

[43] Sosdian S, Rosenthal Y (2009). Deep-sea temperature and ice volume changes across the Pliocene-Pleistocene climate transitions. Science.

[44] Detlef H (2018). Sea ice dynamics across the Mid-Pleistocene transition in the Bering Sea. Nat. Commun..

[45] Martínez-Garcia A (2011). Southern Ocean dust–climate coupling over the past four million years. Nature.

[46] Ginés A, Ginés J (1974). Consideraciones sobre los mecanismos de fosilización de la Cova de sa Bassa Blanca y su paralelismo con formaciones marinas del Cuaternario. Bol. Soc. Hist. Nat. Baleares.

[47] Boop LM (2014). Groundwater geochemistry observations in littoral caves of Mallorca (western Mediterranean): Implications for deposition of phreatic overgrowths on speleothems. Int. J. Speleol..

[48] Csoma AE, Goldstein RH, Pomar L (2006). Pleistocene speleothems of Mallorca: Implications for palaeoclimate and carbonate diagenesis in mixing zones. Sedimentology.

[49] Decker DD, Polyak VJ, Asmerom Y, Lachniet MS (2018). U-Pb dating of cave spar: A new shallow crust landscape evolution tool. Tectonics.

[50] Ludwig KR, Titterington DM (1994). Calculation of ^230^Th/U isochrons, ages, and errors. Geochim. Cosmochim. Acta.

[51] Ludwig, K. R. *User's manual for Isoplot 3.75: A geochronological toolkit for Microsoft Excel.* http://www.bgc.org/isoplot_etc/isoplot/isoplot3_75-4_15manual.pdf (Berkeley Geochronology Center Special Publication 5, 2012).

[52] Kendall RA, Mitrovica JX, Milne GA (2005). On post-glacial sea level — II. Numerical formulation and comparative results on spherically symmetric models. Geophys. J. Int..

[53] Dalca AV (2013). On postglacial sea level — III. Incorporating sedimen redistribution. Geophys. J. Int..

[54] Peltier WR, Argus DF, Drummond R (2015). Space geodesy constrains ice age terminal deglaciation: The global ICE-6G_C (VM5a) model. J. Geophys. Res..

[55] Dziewonski AM, Anderson DL (1981). Preliminary reference Earth model. Phys. Earth Planet. Int..

[56] Sàbat F, Gelabert B, Rodríguez-Perea A, Giménez J (2011). Geological structure and evolution of Majorca: Implications for the origin of the Western Mediterranean. Tectonophysics.

[57] Gelabert B, Sàbat F, Rodríguez-Perea A (1992). A structural outline of the Serra de Tramuntana of Mallorca (Balearic Islands). Tectonophysics.

[58] Fornós JJ (2002). Phreatic overgrowths on speleothems: a useful tool in structural geology in littoral karstic landscapes. The example of eastern Mallorca (Balearic Islands). Geodin. Acta.

[59] Austermann J (2015). The impact of dynamic topography change on Antarctic ice sheet stability during the mid-Pliocene warm period. Geology.

[60] Creveling JR, Mitrovica JX, Hay CC, Austermann J, Kopp RE (2015). Revisiting tectonic corrections applied to Pleistocene sea-level highstands. Quatern. Sci. Rev..

[61] Ferrier KL, Mitrovica JX, Giosan L, Clift PD (2015). Sea-level responses to erosion and deposition of sediment in the Indus River basin and the Arabian Sea. Earth Planet. Sci. Lett..

[62] Blanc P-L (2006). Improved modelling of the Messinian salinity crisis and conceptual implications. Palaeogeogr. Palaeoclimatol. Palaeoecol..

[63] Ryan WBF (2009). Decoding the Mediterranean salinity crisis. Sedimentology.

[64] Mascle G, Mascle J (2019). The Messinian salinity legacy: 50 years later. Mediterranean Geosci. Rev..

