# Author Correction: Sea-level stands from the Western Mediterranean over the past 6.5 million years

**DOI:** 10.1038/s41598-021-85719-z

**Published:** 2021-03-17

**Authors:** Oana A. Dumitru, Jacqueline Austermann, Victor J. Polyak, Joan J. Fornós, Yemane Asmerom, Joaquín Ginés, Angel Ginés, Bogdan P. Onac

**Affiliations:** 1 Karst Research Group, School of Geosciences, University of South Florida, 4202 E. Fowler Ave., NES 107, Tampa, FL 33620 USA; 2 Biology and Paleo Environment Division, Columbia University, Lamont‑Doherty Earth Observatory, Palisades, NY 10964 USA; 3 Department of Earth and Environmental Sciences, Columbia University, Lamont‑Doherty Earth Observatory, Palisades, NY 10964 USA; 4 Department of Earth and Planetary Sciences, University of New Mexico, Albuquerque, NM 87131 USA; 5 Earth Sciences Research Group, Universitat de Les Illes Balears, Ctra. Valldemossa km 7.5, 07122 Palma, Mallorca Spain; 6 Emil G. Racoviţă Institute, Babeş-Bolyai University, Clinicilor 5‑7, 400006 Cluj‑Napoca, Romania

Correction to: *Scientific Reports* 10.1038/s41598-020-80025-6, published online 21 January 2021

The Acknowledgements section in this Article is incomplete.

“We thank the owners and administration of Coves d’Artà and Coves del Drac for granting permission and offering logistic support during the field research conducted for this study. B.P.O. and V.J.P. are funded by a collaborative NSF Grant (AGS 1602670 and 1602685). Additional research costs were covered by NSF Grant (EAR 0326902) to Y.A. and V.J.P. and MINECO CGL2013-48441-P and CGL2016-79246-P grants to J.J.F. O.A.D. received student research grants from the Cave Research Foundation and the Fred L. and Helen M. Tharp Endowed Scholarship (School of Geosciences, University of South Florida). J.A. and O.A.D. thank the PALSEA working group for facilitating discussions at regular meetings. J.A. thanks the Vetlesen Foundation for support. Serge Berné is thanked for his constructive review that improved our manuscript.”

should read:

“We thank the owners and administration of Coves d’Artà and Coves del Drac for granting permission and offering logistic support during the field research conducted for this study. B.P.O. and V.J.P. are funded by a collaborative NSF Grant (AGS 1602670 and 1602685). Additional research costs were covered by NSF Grant (EAR 0326902) to Y.A. and V.J.P. and FEDER/Ministerio de Ciencia, Innovación y Universidades – Agencia Estatal de Investigación CGL2013-48441-P and CGL2016-79246-P grants to J.J.F. O.A.D. received student research grants from the Cave Research Foundation and the Fred L. and Helen M. Tharp Endowed Scholarship (School of Geosciences, University of South Florida). J.A. and O.A.D. thank the PALSEA working group for facilitating discussions at regular meetings. J.A. thanks the Vetlesen Foundation for support. Serge Berné is thanked for his constructive review that improved our manuscript.”

