# Supplementary material for: Sea-level stands from the Western Mediterranean over the past 6.5 million years
Source: Sci Rep. 2021 Jan 21;11:261. doi: 10.1038/s41598-020-80025-6 (PMC7820252; doi:10.1038/s41598-020-80025-6)
Supplement: Supplementary file 1 — Supplementary Legend. [file 41598_2020_80025_MOESM1_ESM.docx]

**Sea-level stands from the Western Mediterranean over the past 6.5 million years**

Oana A. Dumitru^1,2^, Jacqueline Austermann^3^, Victor J. Polyak^4^, Joan J. Fornós^5^, Yemane Asmerom^4^, Joaquín Ginés^5^, Angel Ginés^5^ & Bogdan P. Onac^1,6,^*

^1^Karst Research Group, School of Geosciences, University of South Florida, 4202 E. Fowler Ave., NES 107, Tampa, FL 33620, USA

^2^Biology and Paleo Environment Division, Columbia University, Lamont-Doherty Earth Observatory, Palisades, NY 10964, USA

^3^Department of Earth and Environmental Sciences, Columbia University, Lamont-Doherty Earth Observatory, Palisades, NY 10964, USA

^4^Department of Earth and Planetary Sciences, University of New Mexico, Albuquerque, NM 87131, USA

^5^Earth Sciences Research Group, Universitat de les Illes Balears, Ctra. Valldemossa km 7.5, 07122 Palma (Mallorca), Spain

^6^Emil G. Racoviță Institute, Babeș-Bolyai University, Clinicilor 5-7, 400006, Cluj-Napoca, Romania

*Correspondence and requests for materials should be addressed to: bonac@usf.edu

**Supplementary Information**

**Supplementary** **Table S1.** U-Th-Pb measurement results of Mallorca phreatic overgrowth on speleothems
